# Clinical features and outcome of corneal opacity associated with congenital glaucoma

**DOI:** 10.1186/s12886-018-0865-4

**Published:** 2018-08-02

**Authors:** Yu Jeong Kim, Jin Wook Jeoung, Mee Kum Kim, Ki Ho Park, Young Suk Yu, Joo Youn Oh

**Affiliations:** 1 Laboratory of Ocular Regenerative Medicine and Immunology, Seoul Artificial Eye Center, Seoul National University Hospital Biomedical Research Institute, Seoul, Korea; 2 Department of Ophthalmology, Hanyang University Hospital, Hanyang University College of Medicine, Seoul, Korea; 3 Department of Ophthalmology, Seoul National University Hospital, 101 Daehak-ro, Jongno-gu, Seoul, 110-744 Korea

**Keywords:** Congenital glaucoma, Corneal opacity, Penetrating keratoplasty

## Abstract

**Background:**

To investigate the clinical features of corneal opacity and the surgical outcome of penetrating keratoplasty (PK) in eyes with congenital glaucoma.

**Methods:**

A retrospective review was made of the records from 320 eyes of 193 patients who were diagnosed with congenital glaucoma between January 1981 and January 2016. Anterior segment photographs at disease presentation were examined for the presence and severity of corneal opacity. Data on patient demographics, intraocular pressure (IOP), ocular and systemic comorbidities, ocular surgery and its outcome were collected.

**Results:**

Overall, corneal opacification was observed in 248 of 320 eyes (77.5%). Out of 248 eyes with corneal opacification, 53 eyes had Haab striae alone, and 195 eyes presented with either nebulomacular corneal opacity (128 eyes, iris details visible through opacity) or leukomatous corneal opacity (67 eyes, iris details invisible through opacity). In 12 eyes with severe leukomatous corneal opacity, PK was performed at the mean age of 18.6 months (range 4–57 months). The grafts failed in 6 eyes (50%) due to endothelial rejection (4 eyes) or graft infection (2 eyes) during the mean 80.6 months of follow-up (range 15–228 months). The median survival time was 36 months. The graft failure was significantly associated with smaller corneal diameter at the time of surgery, but not with the age, IOP, combined aniridia, simultaneous glaucoma or lens surgery.

**Conclusion:**

Congenital glaucoma was combined with corneal opacity in 77.5%. The corneal transplant survival was 50% in eyes with congenital glaucoma and total corneal opacity.

## Background

Congenital glaucoma (CG) is a rare disease with the incidence largely varying upon the ethnicity [1–3]. Studies reported that the annual incidence of CG was 2.85 to 5.41 in 100,000 live births in Caucasian populations [3, 4], whereas it was higher in South Asian children [3]. Despite its rarity, CG is often associated with poor visual and functional outcome [5, 6], and it is estimated that glaucoma is responsible for 4–18% of childhood blindness [3, 6–8]. Hence, early detection of the disease and proper treatment are necessary for the vision in pediatric patients with CG.

The classical triad of symptoms in congenital glaucoma includes epiphora, photophobia, and blepharospasm [1]. However, the most common signs first recognized by parents or doctors are corneal abnormalities such as corneal enlargement (buphthalmos) due to increased intraocular pressure (IOP) or corneal clouding as a result of Descemet’s membrane tears (Haab striae) or stromal edema [9–13]. In addition, as it is one form of developmental anomaly of anterior segment, CG is often combined with corneal opacity as a sequel to anterior segment dysgenesis. In these cases, corneal opacity can lead to sensory deprivation amblyopia, and the visual outcome can be poor despite optimal control of IOP. One study reported that corneal opacity along with anisometropia was responsible for vision loss in 50% of childhood glaucoma patients [14]. Therefore, evaluation for corneal opacity and its management are critical for early diagnosis of the disease and the favorable outcome in patients with CG.

We performed this study to evaluate the incidence and clinical characteristics of corneal opacity combined with CG and to investigate the surgical outcome of penetrating keratoplasty (PK) and clinical factors affecting the outcome in eyes with CG.

## Methods

This retrospective study was approved by the Institutional Review Board (IRB No. 1706–094-860). Medical records were reviewed for 320 eyes of 193 Korean patients who were diagnosed with CG between January 1981 and January 2016.

**Fig. 1:**
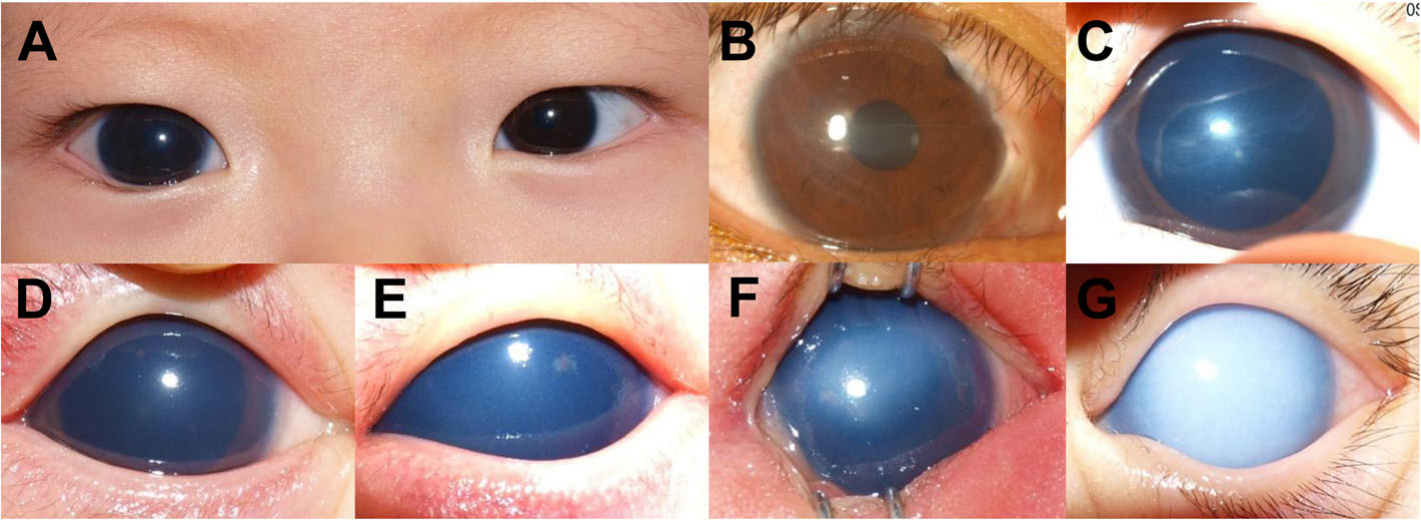
Representative photographs of corneal abnormalities associated with congenital glaucoma. **a** Enlarged cornea (buphthalmos) in the right eye with congenital glaucoma. **b**, **c** Horizontal lines of Haab striae are present in the cornea. **d** Grade 1 corneal opacity. Minimal and superficial opacity is observed. **e** Grade 2 corneal stromal opacity. Both anterior chamber and iris are well-visible despite the opacity. **f** Grade 3 corneal stromal opacity. The pupil is still visible but iris details difficult to see through the opacity. **g** Grade 4 corneal stromal opacity. Pupil is invisible due to total stromal opacity of the cornea

The diagnosis of CG was taken as recorded in the charts. The diagnosis was usually made on the basis of two or more of the following ocular findings: elevated IOP (> 21 mmHg), buphthalmos (enlarged corneal diameter, Fig. 1a), Haab striae, corneal stromal edema, and glaucomatous optic disc change which had been present at birth or shortly after birth. Glaucoma of childhood or juvenile onset was excluded.

Data collected were the patient demographics, laterality of disease, IOP, corneal diameter, ocular comorbidities (aniridia, Peters anomaly, cataract, posterior segment anomalies), systemic abnormalities (Sturge-Weber syndrome, neurofibromatosis, congenital heart disease, TORCH positivity, cerebral palsy, Wilms tumor, chromosomal anomaly), ocular surgery (PK, glaucoma surgery, lens extraction), and last-recorded visual acuity.

In addition, anterior segment photographs at first presentation of a patient were reviewed for the presence of corneal opacity, and the corneal findings were classified as follows: 1) completely clear cornea, 2) Haab striae only (Fig. 1b, c), and 3) corneal opacity. The severity of corneal opacity was further graded based on both four-stage system and two-scale system. The four-stage system followed the corneal opacity scoring system suggested by Gupta et al. [15, 16] with modification: stage 1 = minimal opacity (Fig. 1d), stage 2 = moderate stromal opacity (anterior chamber and iris both well visualized, Fig. 1e), stage 3 = significant stromal opacity (pupil visible with haze, Fig. 1f), and stage 4 = intense stromal opacity (pupil invisible, Fig. 1g). The two-scale system was composed of nebulomacular corneal opacity (iris details visible through opacity) and leukomatous corneal opacity (unable to visualize iris details through opacity). Overall, nebulomacular corneal opacity included stage 1 and 2 opacities, and leukomatous corneal opacity comprised stage 3 and 4 opacities.

The data were additionally analyzed in 12 eyes of 10 CG patients with stage 4 corneal opacity who underwent PK. The graft failure was determined when corneal clarity was irreversibly lost under slit-lamp examination, and the graft survival time was defined as the period from PK to the day when the graft failure was first noted. When multiple PKs were performed in a patient, the first surgery was taken for analysis. The association of age, IOP at the time of surgery, corneal diameter, donor/recipient trephine sizes, difference of sizes between donor and recipient trephines, presence of ocular comorbidities, and concurrent lens or glaucoma surgeries with corneal graft outcome was evaluated.

Data were presented as mean ± SD. The GraphPad Software (GraphPad Prism, La Jolla, CA) was used for statistical analysis. Comparison of quantitative variables between two groups was made by using Student *t* test. The correlation between IOP and the severity of corneal opacity was tested by using Pearson r coefficient and two-tailed *P* value. Survival analysis was performed using the Kaplan–Meier method to estimate the median time to graft failure. The association with the surgical outcome and each clinical factor was analyzed using Fisher’s exact test for qualitative variables and two-tailed Student *t* test for quantitative variables. A *P* value < 0.05 was considered statistically significant.

## Results

### Demographical, ocular and systemic features of CG patients

The demographical, ocular and systemic findings of patients are summarized in Table 1. Of a total 320 eyes in 193 Korean patients with CG, 116 patients (60.1%) were male and 77 (39.9%) were female. The disease was bilateral in 127 patients (65.8%) and unilateral in 66 patients (34.2%) (Right: Left = 26: 40).

**Table 1:** Demographics and clinical feature of patients with congenital glaucoma (193 patients, 320 eyes)

Clinical characteristics		No of patients	%
Gender			
Female		77	39.9
Male		116	60.1
Laterality			
Bilateral		127	65.8
Unilateral	Rt	26	13.5
	Lt	40	20.7
IOP of involved eyes at presentation (mmHg)			
Involved eyes (range)		28.3 ± 9.3 (9.0–54.7)	
Uninvolved eyes (range)		12.9 ± 2.9 (6.0–29.0)	
*P* value		< 0.0001	
Corneal diameter (Horizontal, mm)			
Involved eyes (range)		12.5 ± 1.2 (7.0–16.5)	
Uninvolved eyes (range))		10.9 ± 0.9 (9.0–11.5)	
*P* value		< 0.0001	
Systemic comorbidity		36	18.7
Sturge-Weber syndrome		25	12.9
Congenital heart disease		4	2.1
Neurofibromatosis		2	1.0
TORCH		2	1.0
Cerebral palsy		1	0.5
Wilms tumor		1	0.5
Chromosomal abnormality		1	0.5
Ocular comorbidity		38	19.7
Aniridia		14	7.3
Peters anomaly		7	3.6
Cataract		4	2.1
Posterior segment anomaly		13	6.7

The IOP at first presentation was 28.3 ± 9.3 mmHg (range 9.0–54.7 mmHg) in eyes with CG and 12.9 ± 2.9 mmHg (range 6.0–29.0 mmHg) in eyes without CG (*P* <  0.0001). The horizontal corneal diameter was 12.5 ± 1.2 mm (range 7.0–16.5 mm) at presentation in eyes with CG, which was significantly larger than that of the eye without CG (10.9 ± 0.9 mm, range 9.0–11.5 mm) in the same population (*P* <  0.0001). Thirty-six patients (18.7%) had systemic diseases. Sturge-Weber syndrome was the most common anomaly combined with CG (*n* = 25, 12.9%). Other systemic comorbidities included congenital heart disease (*n* = 4, 2.1%), neurofibromatosis (n = 2, 1.0%), TORCH positivity (n = 2, 1.0%), cerebral palsy (*n* = 1, 0.5%), Wilms tumor (n = 1, 0.5%), and chromosomal anomaly (n = 1, 0.5%).

Congenital ocular comorbidities were observed in 38 patients (19.7%) and included aniridia (*n* = 14, 7.3%), Peters anomaly (*n* = 7, 3.6%), congenital cataract (n = 4, 2.1%), and posterior segment anomaly (*n* = 13, 6.7%).

### Corneal opacification combined with CG

Overall, corneal opacification was observed in 248 of 320 eyes with CG (77.5%), while the cornea was completely clear in 72 eyes (22.5%) (Table 2). Among 248 eyes with corneal opacification, 53 (16.6%) had only Haab striae in the cornea (Fig. 1b, c), and 195 (60.9%) had corneal opacity in the presence or absence of Haab striae (Table 2). The severity of corneal opacity in 195 CG eyes combined with corneal opacity was as follows: grade 1 in 68 eyes (21.2%, Fig. 1d), grade 2 in 60 eyes (18.8%, Fig. 1e), grade 3 in 31 eyes (9.7%, Fig. 1f), and grade 4 in 36 eyes (11.2%, Fig. 1g). When classified based on the two-scale system, nebulomacular corneal opacity was observed in 128 eyes (40%), and leukomatous corneal opacity in 67 eyes (20.9%) (Table 2). There was no significant correlation between the severity of corneal opacity and IOP (*R* = 0.012, *P* = 0.887).

**Table 2:** Keratopathy combined with congenital glaucoma (320 eyes)

Corneal findings		No of eyes (%)	IOP (mmHg, range)
Completely clear cornea		72 (22.5)	24.0 ± 7.8 (11.0–42.1)
Haab striae only		53 (16.6)	24.3 ± 9.7 (11.0–46.0)
Corneal opacification ± Haab striae		195 (60.9)	28.3 ± 9.9 (9.0–54.7)
Nebulomacular opacity	Grade 1	68 (21.2)	27.1 ± 10.6 (9.0–50.0)
	Grade 2	60 (18.8)	28.5 ± 10.3 (13.0–54.7)
Leukomatous opacity	Grade 3	31 (9.7)	30.5 ± 7.6 (17.0–43.0)
	Grade 4	36 (11.2)	28.6 ± 10.1 (9.0–47.0)

*IOP* Intraocular pressure

### Surgical outcome of corneal transplantation in eyes with CG

We additionally analyzed the data of 12 eyes (10 patients) that underwent PK because of non-resolving grade 4 leukomatous corneal stromal opacity. Eight patients had PK in one eye, and 2 underwent PK in both eyes. The demographical, clinical and surgical data are shown in Table 3. The patients included 4 female and 6 male. CG was bilateral in 2 patients and unilateral in 8 patients (Right: Left = 2: 6). The age at time of PK was 18.6 ± 17.8 months (range 4 to 57 months). Aniridia was combined in 7 eyes (5 with total aniridia and 2 with partial aniridia), Peters anomaly in 5 eyes, cataract in 1 eye, and posterior segment abnormality (retinopathy of prematurity) in 1 eye.

**Table 3:** Demographics, clinical and surgical features of patients with penetrating keratoplasty (*n* = 10, 12 eyes)

Clinical or surgical parameters		
Gender (No of patients, %)		
Female		4 (40%)
Male		6 (60%)
Laterality (No of patients, %)		
Bilateral		2 (20%)
Unilateral	Rt	2 (20%)
	Lt	6 (60%)
Age at time of surgery (months, range)		18.6 ± 17.8 (4–57)
The postoperative follow-up (months, range)		80.6 ± 77.8 (15–228)
Systemic comorbidity (No of patients, %)		
Congenital heart disease		1 (10%)
Ocular comorbidity (No of eyes, %)		
Aniridia	Total	5 (41.7%) 2 (16.7%)
	Partial	
Peters anomaly		5 (41.7%)
Cataract		1 (8.3%)
Retinopathy of prematurity		1 (8.3%)
IOP at time of surgery (mmHg, range)		35.5 ± 9.3 (20.0–47.8)
Corneal diameter (mm, range)		
Horizontal		11.7 ± 1.8 (9.5–15.0)
Vertical		10.7 ± 1.7 (9.0–14.0)
Trephine diameter (mm, range)		
Recipient		6.9 ± 0.8 (6.0–8.0)
Donor		7.5 ± 0.7 (6.5–8.5)
Concurrent surgery (No of eyes, %)		
Glaucoma surgery (valve surgery)		4 (33.3%)
Lens extraction		3 (25%)

*IOP* Intraocular pressure

The IOP at time of surgery was 35.5 ± 9.3 mmHg (range 20.0–47.8 mmHg), and all eyes were treated with multiple anti-glaucoma medications. The corneal diameters measured at the time of PK were 11.7 ± 1.8 mm (range 9.5–15.0 mm) horizontally and 10.7 ± 1.7 mm (range 9.0–14.0 mm) vertically. The sizes of trephines used for PK were 6.9 ± 0.8 mm (range 6.0–8.0 mm) for recipient beds and 7.5 ± 0.7 mm (range 6.5–8.5 mm) for donor buttons. In 4 eyes, glaucoma valve implantation surgery was simultaneously performed with PK, and in 3 eyes, lens extraction was done in an open-sky manner during PK.

**Fig. 2:**
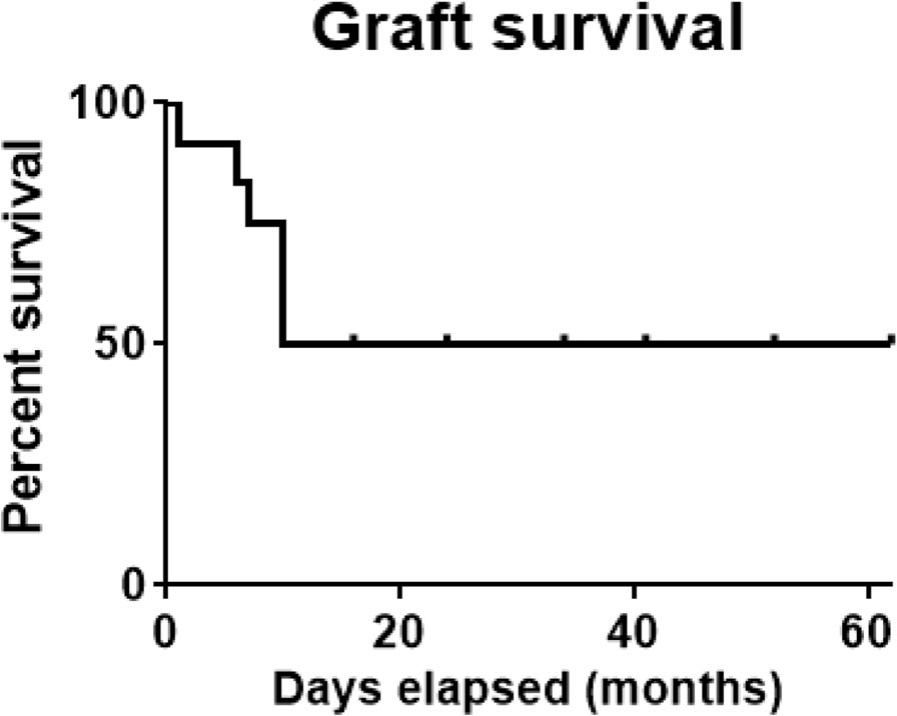
The Kaplan-Meier survival curve of primary penetrating keratoplasty in patients with congenital glaucoma. Out of 12 corneal grafts, 6 failed within 10 months after surgery (range 1–10 months) during the mean 80.6 months of follow-up (range 15–228 months). One year graft survival probability was 50%, and the median survival time of the grafts was 36 months

Over the mean 80.6 ± 77.8 months of follow-up (range 15–228 months), the corneal grafts failed in 6 eyes (50%) whereas they survived in 6 eyes (50%) (Fig. 2). The mean time to graft failure after PK was 7.3 ± 3.5 months (range 1–10 months) in patients with graft failure (Fig. 2), and the mean postoperative follow-up period in those with graft success was 38.2 ± 17.2 months (range 16–62 months). The median survival time of the grafts was 36 months. Out of 6 eyes with graft failure, 4 were caused by endothelial rejection, and 2 were due to graft infection. Among 6 eyes with graft failure, a repeat PK was performed in 4 eyes, 3 of which (66.7%) had the graft failure after regrafting (2 eyes with endothelial rejection and 1 with graft infection), an indication that the outcome of a repeat PK was worse than that of the first surgery. Overall, 4 out of 12 eyes with PK (33.3%) achieved ambulatory vision at the last follow-up as defined by the ability to fixate and follow targets or to count fingers at 3 ft or better [17].

Various clinical and surgical factors were analyzed for their association with corneal graft outcome. Of the factors examined, a smaller diameter of the cornea at the time of surgery was significantly associated with the graft failure. The horizontal corneal diameters were 12.8 ± 1.7 mm in the survival group and 10.4 ± 0.9 mm in the failure group (*P* = 0.0126, Table 4). However, there were no significant associations between the graft survival and other factors such as the age, IOP, the presence of aniridia or Peters anomaly, the difference of sizes between donor and recipient trephines, and simultaneous glaucoma or lens surgery (Table 4).

**Table 4:** Comparison of clinical and surgical factors between the graft survival and failure groups

	Graft survival	Graft failure	*P* value
Aniridia (No. of eyes)			
O	5	2	0.2424
X	1	4	
Peters anomaly (No. of eyes)			
O	4	1	0.2424
X	2	5	
Concurrrent glaucoma surgery (No. of eyes)			
O	1	3	0.5455
X	5	3	
Concurrrent lens extraction (No. of eyes)			1
O	1	2	
X	5	4	
Corneal diameter (horizontal, mm)	12.8 ± 1.7	10.4 ± 0.9	0.0126
Corneal diameter (vertical, mm)	11.7 ± 1.9	9.6 ± 0.9	0.0354
Age at time of surgery (months)	21.7 ± 15.8	15.5 ± 20.7	0.5739
IOP at time of surgery (mmHg)	36.1 ± 12.5	34.9 ± 5.8	0.8373
Trephine gapping^a^ (mm)	0.54 ± 0.3	0.46 ± 0.4	0.8312

aTrephine gapping means the size difference between donor and recipient trephines

## Discussion

Corneal findings are important for suspicion and diagnosis of CG. Studies reported that cloudy cornea and buphthalmos are the most common presenting signs found in over 40% of patients with CG [11, 12]. In our study, 248 of 320 eyes (77.5%) with CG had corneal opacification at disease presentation, whereas 22.5% had completely clear cornea. However, among 77.5% of CG eyes with corneal opacity, 16.6% presented with Haab striae alone and 21.2% had grade 1 minimal opacity. Therefore, visually significant and recognizable corneal opacity (grade 2, 3 or 4 opacity) was observed in 39.7% of the eyes with CG, which is similar to previous reports [11] [12].

Other notable findings of our study are that male was more prevalent than female in CG patients at a ratio of 60.1 to 39.9, and bilateral involvement was more common compared to unilateral involvement at a ratio of 65.8 to 34.2. Overall, 18.7% of CG patients had combined systemic diseases, and the most common systemic comorbidity was Sturge-Weber syndrome which was found in 12.9% of patients with CG. However, Sturge-Weber syndrome was not associated with severe corneal opacity since no patient with Sturge-Weber syndrome underwent PK because of corneal opacity. Neurofibromatosis was combined in two patients (1.0%) in our study. Quaranta et al. [18] reported that neurofibromatosis patients showed gonioscopic findings characteristic of underdevelopment of the iridocorneal angle, suggesting the vulnerability to glaucoma. Of ocular morbidities combined with CG, aniridia was the most frequent (found in 7.3% of CG patients), followed by Peters anomaly (found in 3.6% of CG patients). Combined aniridia and Peters anomaly predisposed patients with CG to severe non-resolving corneal opacity which required PK. Seven out of 14 patients with CG + aniridia and 5 of 7 patients with CG + Peters anomaly underwent PK.

It is generally accepted that the presence of glaucoma and concurrent glaucoma operation during PK are risk factors for poor graft survival in a pediatric population [19, 20]. However, there have just a few case series evaluating the surgical outcome of PK in CG patients, and the results greatly vary upon studies. In our study, the overall graft survival rate was 50% (6 of 12) in eyes with CG during a mean follow-up of 80.6 months, and 33.3% of patients with PK achieved ambulatory vision at the last follow-up. Of note, in the graft failure group, all grafts failed within 10 months (at the mean 7.3 months) after PK. Ariyasu et al. [21] reported that the graft success occurred in 67% of grafts (6 of 9) in eyes with CG during 24 months of follow-up, and ambulatory vision was achieved in 75% of eyes. In a report by Al-Torbak [22], 43 and 17% of corneal grafts survived at 24 and 48 months, respectively after combined PK and Ahmed valve implantation. In our patients, PK was combined with glaucoma valve implantation in 4 eyes, 3 of which had graft failure. By contrast, 3 of 8 eyes without glaucoma surgery had graft failure. This suggests that concurrent glaucoma valve implant surgery might be associated with poor graft outcome, although it did not reach statistical significance due to the small sample size of the current study. Further study with larger sample size would be necessary to confirm the effect of simultaneous glaucoma surgery on the corneal transplant survival.

The only variable affecting the graft outcome was the corneal diameter in our study. The cornea was significantly smaller in the graft failure group, compared to the graft success group. This result is consistent with our previous findings in patients with Peters anomaly or sclerocornea [23]. The proximity of the donor graft to the recipient limbus and more exposure to the host immune system might be related to the high rate of graft failure in eyes with smaller corneas.

Our study is limited by its retrospective nature. Although we here presented the data on corneal opacity determined from the corneal photography in 320 eyes of 193 patients, it was not possible to evaluate other corneal abnormalities such as topographic changes, endothelial cell counts, or hysteresis. Since it was reported that corneal topographic abnormalities were commonly present in CG [13], further prospective study evaluating the cornea from multiple aspects would be helpful to better understand corneal anomalies associated with CG.

## Conclusion

Corneal opacity was a common feature of CG and found in 77.5% of CG patients. The survival rate of corneal allografts in eyes with CG and severe stromal opacity was 50%, and the graft outcome was poorer in eyes with small corneal diameters.

## Abbreviations

CG: Congenital glaucoma
IOP: Intraocular pressure
PK: Penetrating keratoplasty

## References

[1] Ho CL, Walton DS (2004). Primary congenital glaucoma: 2004 update. J Pediatr Ophthalmol Strabismus.

[2] de Luise VP, Anderson DR (1983). Primary infantile glaucoma (congenital glaucoma). Surv Ophthalmol.

[3] Papadopoulos M, Cable N, Rahi J, Khaw PT (2007). The British infantile and childhood Glaucoma (BIG) eye study. Invest Ophthalmol Vis Sci.

[4] Bermejo E, Martinez-Frias ML (1998). Congenital eye malformations: clinical-epidemiological analysis of 1,124,654 consecutive births in Spain. Am J Med Genet.

[5] Dahlmann-Noor A, Tailor V, Bunce C, Abou-Rayyah Y, Adams G, Brookes J, Khaw PT, Papadopoulos M (2017). Quality of life and functional vision in children with Glaucoma. Ophthalmology.

[6] Taylor RH, Ainsworth JR, Evans AR, Levin AV (1999). The epidemiology of pediatric glaucoma: the Toronto experience. J AAPOS.

[7] Franks W, Taylor D (1989). Congenital glaucoma--a preventable cause of blindness. Arch Dis Child.

[8] Gilbert CE, Canovas R, Hagan M, Rao S, Foster A (1993). Causes of childhood blindness: results from west Africa, south India and Chile. Eye (Lond).

[9] Tai TY, Mills MD, Beck AD, Joos KM, Ying GS, Liu C, Piltz-Seymour JR (2006). Central corneal thickness and corneal diameter in patients with childhood glaucoma. J Glaucoma.

[10] Thiagalingam S, Jakobiec FA, Chen T, Michaud N, Colby KA, Walton DS (2009). Corneal anomalies in newborn primary congenital glaucoma. J Pediatr Ophthalmol Strabismus.

[11] Tamcelik N, Atalay E, Bolukbasi S, Capar O, Ozkok A (2014). Demographic features of subjects with congenital glaucoma. Indian J Ophthalmol.

[12] Barsoum-Homsy M, Chevrette L (1986). Incidence and prognosis of childhood glaucoma. A study of 63 cases. Ophthalmology.

[13] Patil B, Tandon R, Sharma N, Verma M, Upadhyay AD, Gupta V, Sihota R (2015). Corneal changes in childhood glaucoma. Ophthalmology.

[14] Robin AL, Quigley HA, Pollack IP, Maumenee AE, Maumenee IH (1979). An analysis of visual acuity, visual fields, and disk cupping in childhood glaucoma. Am J Ophthalmol.

[15] Gupta N, Kalaivani M, Tandon R (2011). Comparison of prognostic value of roper hall and Dua classification systems in acute ocular burns. Br J Ophthalmol.

[16] Choi H, Phillips C, Oh JY, Stock EM, Kim DK, Won JK, Fulcher S. Comprehensive modeling of corneal alkali injury in the rat eye. Curr Eye Res. 2017;42(10):1348–57. 10.1080/02713683.2017.1317817 28636415

[17] Al-Ghamdi A, Al-Rajhi A, Wagoner MD (2007). Primary pediatric keratoplasty: indications, graft survival, and visual outcome. J AAPOS.

[18] Quaranta L, Semeraro F, Turano R, Gandolfo E (2004). Gonioscopic findings in patients with type 1 neurofibromatosis (Von Recklinghausen disease). J Glaucoma.

[19] Huang C, O'Hara M, Mannis MJ (2009). Primary pediatric keratoplasty: indications and outcomes. Cornea.

[20] Karadag R, Chan TC, Azari AA, Nagra PK, Hammersmith KM, Rapuano CJ (2016). Survival of primary penetrating Keratoplasty in children. Am J Ophthalmol.

[21] Ariyasu RG, Silverman J, Irvine JA (1994). Penetrating keratoplasty in infants with congenital glaucoma. Cornea.

[22] Al-Torbak AA (2004). Outcome of combined Ahmed glaucoma valve implant and penetrating keratoplasty in refractory congenital glaucoma with corneal opacity. Cornea.

[23] Kim YW, Choi HJ, Kim MK, Wee WR, Yu YS, Oh JY (2013). Clinical outcome of penetrating keratoplasty in patients 5 years or younger: peters anomaly versus sclerocornea. Cornea.

